# Does a junior doctor focused ‘Bootcamp’ improve the confidence and preparedness of newly appointed ENT registrars to perform their job roles?

**DOI:** 10.1186/s12909-024-05691-w

**Published:** 2024-06-27

**Authors:** Amar Rai, Shivani Shukla, Nikita Mehtani, Vikas Acharya, Neil Tolley

**Affiliations:** 1 Department of Surgery and Cancer, Imperial College Healthcare NHS Trust, London, UK; 2 The School of Clinical Medicine, University of Cambridge, Cambridge, UK; 3 Department of ENT Surgery, Imperial College Healthcare NHS Trust, London, UK; 4 Department of ENT Surgery, St Mary’s Hospital, Praed Street, London, W2 1NY UK

**Keywords:** Surgical training, ENT training, Simulation, Medical education

## Abstract

**Background:**

To assess changes in confidence and preparedness after conducting a 2-day induction bootcamp for novice Ear Nose and Throat (ENT) first year specialty trainee registrars (ST3s) in the United Kingdom (UK). The bootcamp covered common ENT presentations on the ward, and in the elective and emergency settings.

**Methods:**

A total of 32 trainees (ST3 or research fellow) voluntarily registered via an online application form to the Southern ST3 accelerated learning course bootcamp through ENT UK. ENT UK is a membership body that supports ENT trainees throughout their careers. They completed a two-day bootcamp that was hosted at St Mary’s Hospital, London and 10 skills sessions were delivered by either a senior ENT registrar or an ENT consultant. A pre-session questionnaire was distributed to all participants and a post-session questionnaire was provided that assessed the changes in confidence and preparedness of the participants, if any. The responses were scored by a 10-point Likert scale. Only participants who fully completed the pre and post questionnaire were included, which was 29 in total.

**Results:**

Participants self-reported a significant increase in confidence (*p* < 0.001) and preparedness (*p* < 0.001) following the bootcamp course. The greatest improvements in comparison to all other stations were self-preparedness in the rigid bronchoscopy station and self-confidence in the sphenopalatine artery (SPA) ligation station.

**Conclusion:**

The use of a two-day bootcamp improved confidence and preparedness of managing common ENT presentations in the ward, elective and emergency settings for ENT ST3s. It provides a useful adjunct in the acquisition of technical and non-technical skills alongside the traditional surgical apprenticeship. In the future, more work is required to assess the impact of bootcamps on patient outcomes and long-term benefits on trainees’ skill retention and clinical proficiency.

**Supplementary Information:**

The online version contains supplementary material available at 10.1186/s12909-024-05691-w.

## Background

The term ‘bootcamp’ first originated from the United States (US) military to train new recruits [1]. More recently, the intense and compact bootcamp format has been implemented to train medical professionals utilising a combination of simulation training and didactic learning [2]. This training is particularly beneficial to doctors at a period of transition into a new role, which can be demanding due to increased expectations and clinical responsibilities [3]. Furthermore, these transition periods are known to be associated with a decline in patient outcomes, previously termed the so called ‘July effect’ in the US or ‘Killing Season’ in the United Kingdom (UK) [4, 5]. Bootcamps help improve patient care and outcomes by providing doctors with a safe and nonjudgmental setting to practice clinical skills without patient endangerment [4, 6, 7]. This is especially relevant in the post COVID-19 pandemic era, where patient contact may be limited, but hands-on learning remains important for surgeons to learn and train [8].

In the UK, many medical graduates felt underprepared in the management of Ear, Nose and Throat (ENT) conditions as specialist knowledge was not routinely taught at either medical school or in foundation training [1, 8]. Following the completion of two foundation years, trainees who pursue ENT training are required to complete a further two years of core surgical training (CST) before starting their six-year ENT higher surgical training programme, with their first year job role known as a specialty trainee registrar (ST3) [9]. Every year, it is estimated that there are 20–50 new ENT ST3s in the UK, who are expected to treat ENT conditions in emergency and elective situations including operating theatres as well as outpatient clinic [10, 11]. However, the traditional apprenticeship model of surgical education has reported leaving ENT ST3 trainees feeling underconfident and unprepared to undertake their new job role [9, 12]. This is especially significant when considering that some ENT emergencies can be life threatening or uncommon, which minimises learning through repeated clinical exposure [1, 13].

In a move to mitigate these problems, there has been a significant reform in UK surgical training. Apprenticeship training largely depending on formal and informal reports from surgical seniors was replaced in 2007 by the Intercollegiate Surgical Curriculum Programme (ISCP) which is a “General Medical Council (GMC) approved framework for UK surgical training” and falls under the parent body of The Joint Committee on Surgical Training (JCST [11, 12]). An updated 2021 ISCP Otolaryngology report states that it “involves development of competence in diagnostic reasoning, managing uncertainty, dealing with comorbidities, and recognising when another specialty opinion or care is required (as well as developing technical skills in the areas and to the level described in the syllabus…)” [11]. There is a requirement and need for practical proficiency from ENT trainees, as with all surgical specialties, precipitating the relevance of bootcamps in providing simulation based, hands-on and individualised learning is clear.

Subsequently, there has been a significant adoption of bootcamps into Otolaryngology training nationally. Alabi et al. conducted an ENT bootcamp for CSTs focusing on 3 emergency scenarios, namely post-thyroidectomy haematoma, post-tonsillectomy haemorrhage and epistaxis, following which participants reported an increase in confidence [8]. Anmolsingh et al. found an increase in confidence in CST trainees transitioning into ST3 positions in technical and nontechnical skills, however this paper did not examine the preparedness of participants [9]. Preparedness is a psychometric measure that underlies the psychosomatic processes that underly self-validation that reduce negative emotions of stress and anxiety [14]. Engelhardt. et alia showed that under-preparedness among surgical trainees is associated with physician burnout and in a cross-sectional UK study Miller et alia reported that most surgeons felt performance anxiety, often from under-preparedness, negatively impacting their performance and wellbeing [15, 16].

In light of the psychological importance of preparedness in surgical outcomes and the lack of ENT bootcamp studies that assess preparedness, we aimed to evaluate changes in confidence and preparedness after conducting a 2-day induction bootcamp for novice ENT ST3s in the UK. The bootcamp covered common ENT presentations in the ward, elective and emergency settings.

## Methods

### Participants

A total of 32 out of 64 UK ENT ST3 trainees (ST3 or research fellow) registered to the mandatory Southern ST3 accelerated learning course bootcamp through ENT UK [12]. ENT UK is a membership body that supports ENT trainees throughout their careers [13]. Trainees registered for the bootcamp via an online application form. There was a limit of 32 places for this educational bootcamp programme.

### Bootcamp

A two-day bootcamp was hosted at St Mary’s Hospital, London from 12th October 2022–13th October 2022. The bootcamp was designed to improve the skills, knowledge, and confidence of new registrars through 10 skills sessions: (1) Simulated Ward Round (2) Non-technical Skills for Surgeons (NOTSS) (3) Airways (4) Rhinology (5) Management of Bleeding tonsil and adenoid bed (6) Communication station (7) Bronchoscopy (8) Cortical mastoidectomy (9) Thyroidectomy bleed (10) Tracheostomy management and Touch Surgery. An agenda of the structure and description of the day was provided to each delegate (Appendix A).

Each skills session lasted 60 min and was delivered by either a senior ENT registrar or an ENT consultant, who had prior experience in the skills session(s). The sessions included the use of different simulated environments, models and technology to provide a safe environment for trainees to practice.

### Measures

A pre-session questionnaire was distributed to all participants prior to attending the bootcamp to assess confidence in the commencement of ST3 and perceived ability to deal with emergencies on call (Appendix B). Prior practice, confidence and preparedness were assessed for each skills session (Appendix B). All confidence and preparedness questions were recorded via a 10-point Likert scale; a score of zero was equivalent to ‘strongly disagree’ and a score of 10 was equivalent to ‘strongly agree’.

A post-session questionnaire was provided that assessed the confidence and preparedness of the participants following the 10 skills sessions. The questions were scored by a 10-point Likert scale. In addition, the questionnaire included questions about the quality of each session and further opportunity to comment and feedback on the day (Appendix B). The questionnaires were hosted on Google Forms (Google, USA) and the pre and post session responses were paired using Microsoft Excel (Microsoft, USA).

Inclusion criteria: Participants who completed both pre and post session questionnaires were included in this study.

### Statistical methods

Statistical analysis was performed using SPSS V28.0 (IBM Corp., Armonk, NY, USA). The Shapiro-Wilk test was used to test if the data had a normal distribution. A non-parametric data set was identified and the statistical difference between the pre and posted session paired data set was tested using a Wilcoxon signed ranked test. P-value < 0.05 was considered statistically significant.

## Results

### Baseline

A total of 29 participants completed both the pre and post boot camp questionnaires. 27 out of 29 participants were new ST3 trainees and 2 out of 29 were clinical research fellows. Participants were from 11 different National Health Service (NHS deaneries) across the country (Table 1).

**Table 1:** Number of participants (*n* = 29) included in the study from different NHS deaneries across the UK (*n* = 11) and their respective training positions

NHS Deanery	*N*
*East Anglia*	*2*
*KSS*	*2*
*LNR*	*2*
*London*	*7*
*Northern Ireland*	*1*
*Peninsula*	*1*
*Scotland*	*2*
*Severn*	*3*
*Wessex*	*2*
*West Midlands*	*6*
*Other*	*1*
***Level of training***	
*ST3*	*27*
*Research Fellow*	*2*

Participants self-reported a significant increase in confidence (*p* < 0.001) and preparedness (*p* < 0.001) following the bootcamp (Table 2).

**Table 2:** The median (Q1-Q2) preparedness and confidence of ST3 registrars before and after the bootcamp (p value < 0.05)

Prepared and Confidence for ST3 Registrar (*n* = 29)			
	**pre course**	**post course**	**P value**
Confidence	5.0 (4.0–7.0)	8.0 (7.0–9.0)	< 0.0001
Prepared	6.0 (5.0–7.0)	9.0 (8.0–10.0)	< 0.0001

Participants self-reported a significant increase in preparedness to complete inpatient ward rounds (*p* < 0.001), elective procedures in completing a neck access (*p* < 0.001), SPA ligation (*p* < 0.0001), tonsillectomy (*p* < 0.0001), HoloLens (*p* < 0.0001), rigid bronchoscopy (*p* < 0.0001) and cortical mastoidectomy (*p* < 0.0001) (Table 3).

**Table 3:** Self-reported median (Q1-Q2) preparedness and confidence of ST3 registrars before and after the completion of stations (p value < 0.05)

Prepared (*n* = 29)				
Station	Setting	Pre	Post	P value
Ward Rounds	Inpatient	7.0 (6.0–7.0)	8.0 (7.0–9.0)	< 0.001
Neck access	Elective	6.0 (4.0–7.0)	8.0 (7.0–8.0)	< 0.001
	Emergency	4.5 (2.8-5.0)	8.0(6.0–8.0)	< 0.0001
SPA Ligation	Elective	3.0 (1.0–5.0)	7.0 (6.0–8.0)	< 0.0001
	Emergency	2.0 (1.0–3.0)	6.0 (3.0–8.0)	< 0.0001
Tonsillectomy	Elective	7.0 (5.0–8.0)	8.0 (7.0–9.0)	< 0.0001
	Emergency	5.5 (4.0–7.0)	8.0 (7.0–9.0)	< 0.0001
HoloLens	Elective	6.0 (5.0–7.0)	8.0 (7.0–9.0)	< 0.0001
Rigid Bronchoscopy	Elective	3.0 (2.0–5.0)	8.0 (5.0–9.0)	< 0.0001
	Emergency	2.0 (1.0–4.0)	8.0 (5.0–8.0)	< 0.0001
Cortical Mastoidectomy	Elective	3.0 (1.0–4.0)	6.0 (4.0–8.0)	< 0.0001
	Emergency	3.0 (1.0–4.0)	6.0 (4.0–8.0)	< 0.0001
Postoperative Thyroidectomy	Emergency	5.0 (4.0–7.0)	8.0 (8.0–9.0)	< 0.0001
Confidence (*n* = 29)				
Statio	Setting	Pre	Post	P value
Ward Rounds	Inpatient	7.0 (6.0–7.0)	8.0 (7.0–9.0)	< 0.001
Neck access	Elective	5.0 (4.0–7.0)	8.0 (7.0–9.0)	< 0.0001
	Emergency	4.0 (2.0–6.0)	7.0 (6.0–8.0)	< 0.0001
SPA Ligation	Elective	3.0 (1.0–5.0)	7.0 (5.0–8.0)	< 0.0001
	Emergency	2.0 (1.0–4.0)	6.0 (4.0–7.0)	< 0.0001
Tonsillectomy	Elective	7.0 (7.0–8.0)	9.0 (8.0–9.0)	< 0.0001
	Emergency	6.0 (5.0–7.0)	8.0 (7.0–9.0)	< 0.001
HoloLens	Elective	6.0 (5.0–7.0)	8.0 (8.0–8.0)	< 0.001
Rigid Bronchoscopy	Elective	4.0 (2.0–6.0)	7.0 (6.0–9.0)	< 0.001
	Emergency	3.0 (1.0–4.0)	7.0 (4.0–8.0)	< 0.001
Cortical Mastoidectomy	Elective	3.0 (1.0–4.0)	6.0 (4.0–8.0)	< 0.001
	Emergency	2.0 (1.0–4.0)	5.0 (3.0–8.0)	< 0.001
Postoperative Thyroidectomy	Emergency	6.0 (4.0–8.0)	8.0 (8.0–9.0)	< 0.0001
Tracheostomy	Elective	6.0 (5.0–7.0)	7.0 (7.0–9.0)	< 0.001
	Emergency	4.0 (2.0–6.0)	7.0 (5.0–8.0)	< 0.0001

In addition, participants reported a significant increase in preparedness during emergency neck access (*p* < 0.0001), SPA ligation (*p* < 0.0001), tonsillectomy (*p* < 0.0001), rigid bronchoscopy (*p* < 0.0001), cortical mastoidectomy (*p* < 0.0001) and emergency postoperative thyroidectomy(*p* < 0.0001) (Table 3).

Participants self-reported a significant increase in confidence in inpatient ward rounds (*p* < 0.001), elective procedures in completing a front of neck access (*p* < 0.0001), SPA ligation (*p* < 0.001), tonsillectomy (*p* < 0.0001), HoloLens (*p* < 0.001), rigid bronchoscopy (*p* < 0.001), cortical mastoidectomy (*p* < 0.001) and a tracheostomy (*p* < 0.001) (Table 3).

Additionally, participants reported a significant increase in confidence in applying these skills during emergency situations, to complete a front of neck access (*p* < 0.0001), SPA ligation (*p* < 0.0001), tonsillectomy (*p* < 0.001), rigid bronchoscopy (*p* < 0.001), cortical mastoidectomy (*p* < 0.001), postoperative thyroidectomy management (*p* < 0.0001) and emergency tracheostomy (*p* < 0.0001) (Table 3).

**Fig. 1:**
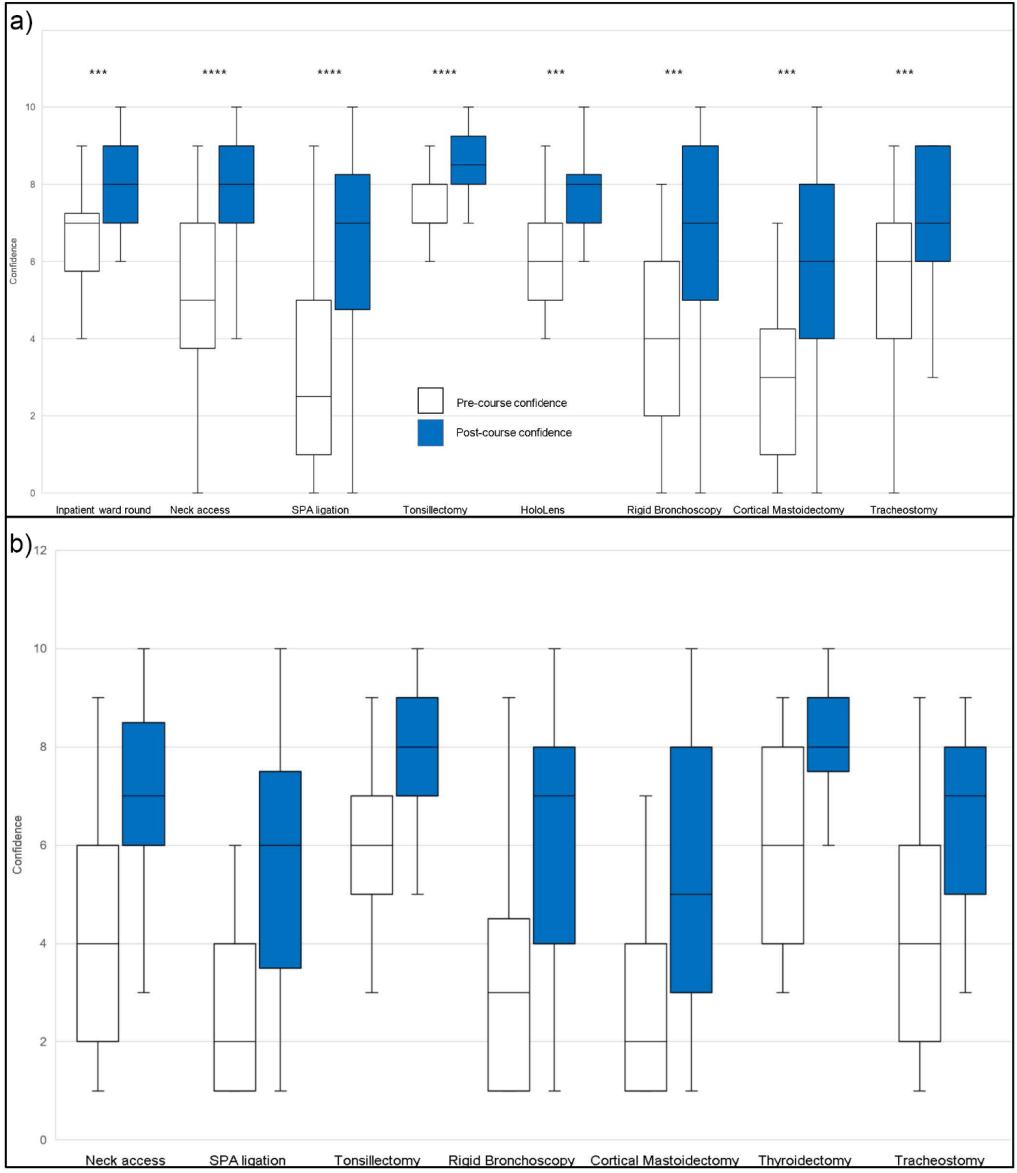
Box and whisker plots showing a significant increase in confidence before and after the bootcamp in common ENT presentations across ward based, elective and emergency settings using the Wilcoxon signed-rank test ( ***p ≤ 0.0001, ****p ≤ 0.0001)

**Fig. 2:**
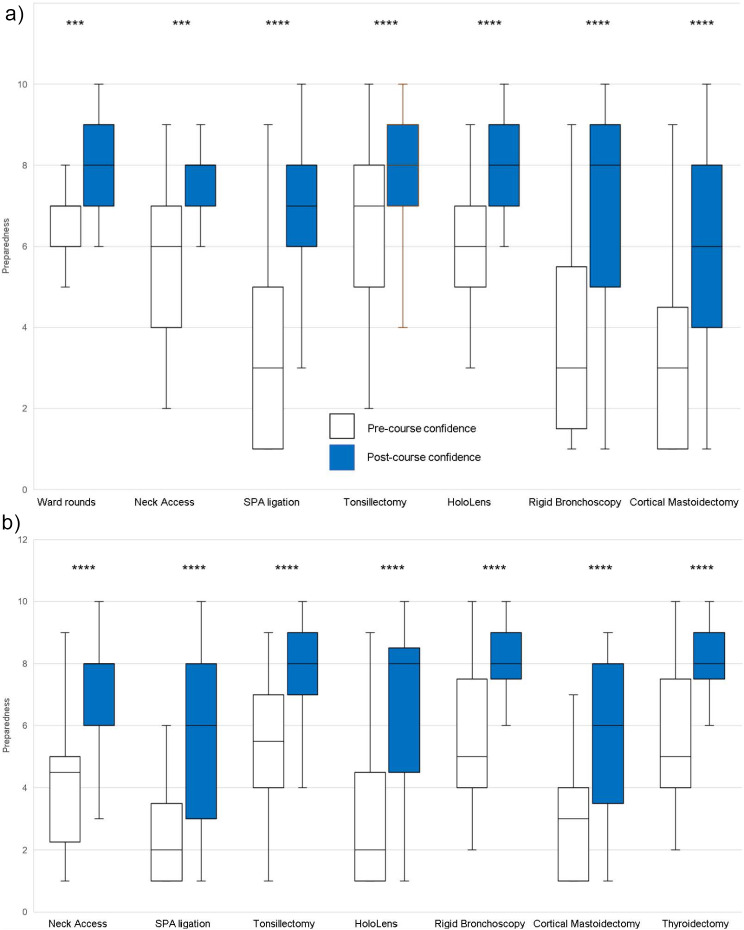
Box and whisker plots showing a significant increase in preparedness before and after the bootcamp in common ENT presentations across ward based, elective and emergency settings using the Wilcoxon signed-rank test ( ***p ≤ 0.0001, ****p ≤ 0.0001)

Overall, significant increases in confidence (Fig. 1) and preparedness (Fig. 2) were observed across ENT stations in a ward round, elective and emergency settings.

## Discussion

The primary purpose of the two day ENT bootcamp is to provide and enhance the skills and knowledge necessary for incoming ENT ST3s as expected by the updated 2021 ISCP curriculum [11]. This study shows that the bootcamp resulted in a statistically significant improvement in both the self-reported preparedness and confidence in the new ENT ST3s (Table 2). New ENT Trainee’s reported a further statistically significant improvement in self-confidence in stations that covered ward rounds and elective skill stations (Fig. 1a) as well as emergency skills stations (Fig. 1b). In addition, participants reported a statistically significant improvement in self-preparedness in stations that covered ward rounds and elective skill stations (Fig. 2a) as well as emergency skills stations (Fig. 2b). This aligns with the prior reported literature of significant improvements in trainee self-confidence following their participation in an ENT boot camp [1, 2, 8, 9, 13, 17–20].

Our data demonstrated that ENT ST3s reported the greatest improvements in self-preparedness in the rigid bronchoscopy station and the greatest improvement in self-confidence in the sphenopalatine artery (SPA) ligation station compared to the other stations (Table 3). While SPA ligation has not been reported in an ENT bootcamp setting, the tracheostomy findings support the literature. For example, Anmolsingh et al. assessed the confidence of ENT higher trainees after a 1-day bootcamp and reported that trainees had a statistically significant improvement (*p* < 0.0001) in their confidence after the completion of technical laryngeal stations [9]. An explanation for the improved preparation and confidence observed by trainees is that participants may be initially unfamiliar with rigid bronchoscopy, which may result in apprehension in completing the task. This is described by Kashat et al., where a 1 day bootcamp was used to teach both anaesthesiology and ENT US residents an introduction to airway knowledge and skills [17]. It was observed that initially 95% of the novice residents were the least comfortable with rigid bronchoscopy as they were not familiar or comfortable with this advanced airway technique [17].

The trainees’ unfamiliarity with the station would result in state of stress, similar to the “fight or flight” response, which may result in fear or avoidance of a task [21]. Caroll et al. correlated preparedness with confidence, where preparedness is described as a state of readiness that is negatively impacted by negative emotions such as fear and under-confidence [14]. For an individual to feel more prepared, they would have to accumulate knowledge and experience to optimise future proficiency and performance, which can be described as confidence [14]. Hence, self-preparation is associated with the self-confidence of a trainee. This supports the notion that a 2-day bootcamp provides ENT ST3s with the knowledge and experience to perform complex technical skills such as rigid bronchoscopy in simulated elective and emergency scenarios (Table 3). Additionally, our bootcamp may reduce the learning curve and negative emotions faced by trainees. This still requires further investigation and assessment.

A high baseline and minimal improvement of self-preparation and self-confidence in inpatient ward rounds (WR), tonsillectomy and elective tracheotomy was observed during our study (Table 3). WRs are the most common responsibility of junior doctors, with a survey conducted of newly graduated first year doctors in Northern Lincolnshire and Goole reporting that 84% conduct a WR alone two or more days per week [22, 23]. This may indicate that trainees were more prepared prior to the course due to familiarity and knowledge processes that enabled them to confidently conduct the WR independently. Alternatively, the results of the tonsillectomy skills station are attributed to the repetitive exposure of the procedure in training [24]. A systematic review by Maruthappu et al. reported that surgeons who experience a higher volume of cases (more experience) have better performance rates, with the exception of the years nearing retirement [25]. Thus, the volume of tonsillectomies performed provides ENT ST3s the experience to minimise the learning curve to perform a tonsillectomy independently [24]. As a result, a high baseline of self-reported preparation of the procedure was observed. It must be noted that trainees still reported a significant improvement (Table 3) in both of these common stations following the bootcamp. This highlights that although ST3s have a high baseline for common ENT stations, the inclusion of the stations in future ENT ST3 focused bootcamps would be beneficial in the training of new ST3s to enhance their capability in the clinical environment even further. Elective tracheostomies are required for the management of patients who require long-term mechanical ventilation. While only a minority of medical schools delivered teaching on tracheostomy, the 2021 ISCP CST curriculum states that ENT trainees should be able to perform and change a tracheostomy as part of “general surgical airway care” [11, 26]. This may mean participants are more likely to be confident with this skill as part of their training as opposed to more specialised ENT procedures, including rigid bronchoscopy.

Our study suggests that ST3 knowledge improved over a two day bootcamp, which along with the literature, provides evidence that knowledge acquisition through bootcamps may help ST3s to transition into their new clinical role more easily [1, 2, 8, 9, 13, 17–20]. Additionally, the inclusion of simulation stations allows ST3s to acquire the technical and non-technical skills expected of ENT ST3s. The literature suggests that bootcamps are a useful adjunct to the traditional apprenticeship training model, as they provide a standardised and equal approach towards the training of ST3s that can integrate the use of performance metrics unlike the variability of clinical exposure [27–29]. There is a gap in the traditional apprenticeship model due to trainees being unable to self-evaluate their progress, which can be mitigated through formally assessed bootcamps [12].

### Limitations

Limitations within the study design exist, for example the lack of a control group for the comparison of results which reduces internal validity. There was no comparison of bootcamp effectiveness to other teaching courses, as thus far, it has only been compared to no teaching in the literature [9]. Additionally, the participants formed a small sample size from a singular location within the UK, meaning caution needs to be taken when extrapolating findings. There is a second national bootcamp also undertaken elsewhere in the UK, and the data from that course could be utilised if measured, in the future.

Previous bootcamps, for example, an anaesthesia based bootcamp by Kazior et al., have used test scores such as The Anaesthesia Knowledge Test as a measure of participants’ technical knowledge [28]. This study measured participants’ change in self-reported confidence and preparedness. A formal assessment of participants’ performance was avoided to prevent stress from impeding learning. Consequently, a key limitation is that the study results are subjective and self-reported confidence and preparedness may not translate to improved bedside clinical proficiency or competence.

Furthermore, trainees were not followed-up over a period of time, which prevents the study from assessing long-term improvements in ST3s confidence and preparedness. For example, Anmolsingh et al. conducted a 12-month post ENT bootcamp interview and found that participants felt safer and more confident, developed technical and non-technical skills and felt a direct improvement in their ST3 clinical performance [9]. Further work is required in a larger sample size to determine if bootcamps translate to improved clinical skill and patient outcomes in the long-term.

In relation to bootcamp stations, the use of artificial mannequins introduces artificial artifacts that impede realism and the whole transfer of skills from simulation to bedside [30]. Malloy et al. carried found within their ENT bootcamp that the mannequin’s mask ventilation seal was being achieved at a low, improper threshold which taught poor technique to real-life patients [31]. Similarly, Malekzadeh et al. reported that mannequin airways were stiffer than patients so lubricant and gentle handling of the laryngoscopy telescopes was needed, adding unrealistic complications to the procedure [32].

Consequently, future studies require a quantitative evaluation of the long-term outcomes of ENT bootcamps in a larger and diverse sample population of surgical trainees. Technical advancement in mannequin design may limit the artifacts introduced in the simulation stations.

## Conclusion

Following participation in a two-day bootcamp, ENT trainees transitioning from core training level to ST3 reported a significant improvement in confidence and preparedness in the management of common ENT presentations in the ward, elective and emergency simulated settings. The use of a two-day bootcamp provides a useful adjunct in the acquisition of technical and non-technical skills alongside the apprenticeship model to train ENT ST3 trainees. Prospectively, further work is required to quantitatively determine whether bootcamps translate to improved clinical skill and patient outcomes in the long-term. Furthermore, bootcamps are unstandardised within the UK which results in a variable quality of training, which can be addressed by involving accreditation and certifying bodies to ensure their adoption nationally [33].

### Electronic supplementary material

Below is the link to the electronic supplementary material.

Supplementary Material 1

Supplementary Material 2

## Data Availability

The datasets used and/or analysed during the current study are available from the corresponding author on reasonable request.
