# Supplementary material for: Does a junior doctor focused ‘Bootcamp’ improve the confidence and preparedness of newly appointed ENT registrars to perform their job roles?
Source: BMC Med Educ. 2024 Jun 27;24:702. doi: 10.1186/s12909-024-05691-w (PMC11212423; doi:10.1186/s12909-024-05691-w)
Supplement: Supplementary file 1 — Supplementary Material 1 [file 12909_2024_5691_MOESM1_ESM.pdf]

Program:

| Day 1: Wednesday 12th October | | |
|---|---|---|
| 08:30 - 08:45 | Candidate Registration & Coffee | |
| 08:45 - 09:00 | Welcome and Introduction | Professor Neil Tolley |
| 09:00 - 10:00 | Skills Session 1 | |
| 10:00 - 10:30 | Lecture: ENT Emergencies 1 | Mr Matthew Rollin |
| 10:30 - 11:00 | Break | |
| 11:00 - 12:00 | Skills Session 2 | |
| 12:00 - 13:00 | Skills Session 3 | |
| 13:00 - 14:00 | Lunch | |
| 14:00 - 15:00 | Skills Session 4 | |
| 15:00 - 16:00 | Skills Session 5 | |
| 16:00 – 16:10 | AOT ENT President | Tharsika Myuran |
| 16:10 – 16:40 | Coffee Break | |
| 16:45 - 17:00 | Debrief and close | Professor Neil Tolley |
| 17:00 onwards | Drinks + Dinner | |

| Day 2: Thursday 13th October | | |
|---|---|---|
| 08:30 – 09:00 | Registration and Coffee | |
| 09:00 - 10:00 | Skills Session 6 | |
| 10:00 - 11:00 | Skills Session 7 | |
| 11:00 - 11:30 | Break | |
| 11:30 - 12:30 | Skills Session 8 | |
| 12:30 - 13:00 | Lecture: ENT Emergencies 2 | Mr Matthew Rollin |
| 13:00 - 14:00 | Lunch | |
| 14:00 - 15:00 | Skills Session 9 | |
| 15:00 - 16:00 | Skills Session 10 | |
| 16:30 - 16:45 | Break | |
| 16:45 - 17:00 | Debrief and close | Professor Neil Tolley |

## Station 1: Simulated Ward Round

Location: Virtual Ward

Description:

A chance to take the lead on a ward round, a skill that will be expected of you as an ENT registrar. It will require skills such as: leadership, tasks management, triage, diagnostics and clinical planning. Participant is the designated registrar with faculty staff playing the role of F2 and a patient. Participant to carry out a ward round and review two patients, construct management plans and communicate this to the patient and team.

## Station 2: Non-Technical Skills for Surgeons (NOTSS)

Location: Virtual operating theatre

Description:

This station is designed to develop your non-technical skills: situational awareness, decision making, communication, teamwork and leadership when in a pressurised simulated environment.

## Station 3: Airway/FONA

Location: Skills lab

Description:

Establishing an emergency surgical airway is a crucial skill for an ENT registrar and might have to be performed without senior supervision. Using porcine models, you will be taught how to perform an emergency tracheostomy and cricothyroidotomy.

## Station 4: Rhinology

Location:Skills lab

Description:

Using bovine models, you will get hands on experience of some of the basic skills required during rhinology surgery. This includes the management of epistaxis and examination under anaesthetic of the nose.

## Station 5: Management of Bleeding tonsil and adenoid bed

Location: Room 2

Description:

Using a custom model, you will learn how to perform a cold steel tonsillectomy, including tonsil ties.

## Station 6: Communication station

Location: Room 1

Description:

Several clinical scenarios which you will require senior advice about. Will allow you to practice your communication skills. As part of this station, you will be testing a new form of communication technology. Created with the Microsoft HoloLens2$^{TM}$ to create a virtual environment where participants had to demonstrate effective communication skills and liaise with senior clinicians on managing deteriorating patients.

## Station 7: Bronchoscopy

Location: Room 5

Description:

The opportunity to assemble and become accustomed with a ridged bronchoscope, then use it on models to perform some basic skills.

## Station 8: Cortical Mastoidectomy

Location: Room 7

Description:

In this station you will have a discussion of the steps of a cortical mastoidectomy, and then have the opportunity to practice using the Voxelman simulator to perform various aspects of a cortical mastoidectomy.

## Station 9: Thyroidectomy bleed

Location: Room 3

Description:

In this station you will learn how to recognise and treat a patient who is having a post tonsillectomy bleed, a potentially fatal complication.

## Station 10: Tracheostomies Management + Touch Surgery

Location: Room 1

Description:

Practical instruction from the Imperial Airway CNS and Consultants to educate about common problems associated with Tracheostomy utilizing the application Touch Surgery. This with allow trainees to acquire knowledge on how to advise ITU colleagues and problem solve the common scenarios seen in tracheostomy patients.
