# Supplementary material for: Does a junior doctor focused ‘Bootcamp’ improve the confidence and preparedness of newly appointed ENT registrars to perform their job roles?
Source: BMC Med Educ. 2024 Jun 27;24:702. doi: 10.1186/s12909-024-05691-w (PMC11212423; doi:10.1186/s12909-024-05691-w)
Supplement: Supplementary file 2 — Supplementary Material 2 [file 12909_2024_5691_MOESM2_ESM.pdf]

ENT Southern ST3 Accelerated Learning Course:

PRE SESSION-QUESTIONNAIRE

Name for Certificate	
Email Address for Certificate	
Deanery / Hospital	
HST ST3 / Run Through Trainee / Other	
**Prior to attending this bootcamp teaching event, please rate your confidence and preparedness for starting as an ST3 registrar:**	
I felt confident about starting my ST3 registrar training	Likert Scale 0-10: Strongly Disagree to Strongly Agree. 5=Neutral.
I felt confident to deal with emergencies on-call as the ST3 registrar	Likert Scale 0-10: Strongly Disagree to Strongly Agree. 5=Neutral.
I felt prepared to generally and overall undertake my job role as an ST3 registrar	Likert Scale 0-10: Strongly Disagree to Strongly Agree. 5=Neutral.
I felt prepared to be able to perform my day to day duties as an ST3 registrar	Likert Scale 0-10: Strongly Disagree to Strongly Agree. 5=Neutral.
**STATION 1 (Rigid Ventilating Bronchoscopy)**	
Have you practiced this in a simulated environment before?	Y / N
I am confident to perform a rigid bronchoscopy on a real patient in an elective setting	Likert Scale 0-10: Strongly Disagree to Strongly Agree. 5=Neutral.
I am confident to perform a rigid bronchoscopy on a real patient in an emergency situation/setting	Likert Scale 0-10: Strongly Disagree to Strongly Agree. 5=Neutral.
I feel prepared enough to perform this procedure electively in a clinical setting	Likert Scale 0-10: Strongly Disagree to Strongly Agree. 5=Neutral.
I feel prepared enough to perform this procedure acutely when on-call in an emergency situation	Likert Scale 0-10: Strongly Disagree to Strongly Agree. 5=Neutral.
**STATION 2 (Mastoidectomy)**	
Have you practiced this in a simulated environment before?	Y / N
I am confident to perform a cortical mastoidectomy on a real patient in an elective setting	Likert Scale 0-10: Strongly Disagree to Strongly Agree. 5=Neutral.
I am confident to perform a cortical mastoidectomy on a real patient in an emergency situation/setting	Likert Scale 0-10: Strongly Disagree to Strongly Agree. 5=Neutral.
I feel prepared enough to perform this procedure electively in a clinical setting	Likert Scale 0-10: Strongly Disagree to Strongly Agree. 5=Neutral.
I feel prepared enough to perform this procedure acutely when on-call in an emergency situation	Likert Scale 0-10: Strongly Disagree to Strongly Agree. 5=Neutral.
**STATION 3 (leading a WR)**	
I am confident to lead an inpatient ward	Likert Scale 0-10: Strongly Disagree to

round in a hospital setting	Strongly Agree. 5=Neutral.
I feel prepared enough to lead ward rounds on inpatients	Likert Scale 0-10: Strongly Disagree to Strongly Agree. 5=Neutral.
Have you been the lead clinician for inpatient ward rounds before?	Y / N
Have you practiced this in a simulated environment before?	Y / N
**STATION 4 (NOTSS)**	
Have you practiced this in a simulated environment before?	Y / N
**STATION 5 (Hololens)**	
Have you practiced this in a simulated environment before?	Y / N
I am confident to manage acute ENT conditions in an elective setting	Likert Scale 0-10: Strongly Disagree to Strongly Agree. 5=Neutral.
I feel prepared enough to manage acute ENT conditions electively in a clinical setting	Likert Scale 0-10: Strongly Disagree to Strongly Agree. 5=Neutral.
**STATION 6 (SPA ligation)**	
Have you practiced this in a simulated environment before?	Y / N
I am confident to perform an SPA ligation on a real patient in an elective setting	Likert Scale 0-10: Strongly Disagree to Strongly Agree. 5=Neutral.
I am confident to perform SPA ligation on a real patient in an emergency situation/setting	Likert Scale 0-10: Strongly Disagree to Strongly Agree. 5=Neutral.
I feel prepared enough to perform this procedure electively in a clinical setting	Likert Scale 0-10: Strongly Disagree to Strongly Agree. 5=Neutral.
I feel prepared enough to perform this procedure acutely when on-call in an emergency situation	Likert Scale 0-10: Strongly Disagree to Strongly Agree. 5=Neutral.
**STATION 7 (Tracheostomy)**	
Have you practiced this in a simulated environment before?	Y / N
I am confident to perform a surgical tracheostomy on a real patient in an elective setting	Likert Scale 0-10: Strongly Disagree to Strongly Agree. 5=Neutral.
I am confident to perform a surgical tracheostomy on a real patient in an emergency situation/setting	Likert Scale 0-10: Strongly Disagree to Strongly Agree. 5=Neutral.
I feel prepared enough to perform this procedure electively in a clinical setting	Likert Scale 0-10: Strongly Disagree to Strongly Agree. 5=Neutral.
I feel prepared enough to perform this procedure acutely when on-call in an emergency situation	Likert Scale 0-10: Strongly Disagree to Strongly Agree. 5=Neutral.
**STATION 8 (Post Thyroidectomy bleed)**	
Have you practiced this in a simulated environment before?	Y / N
I am confident to manage postoperative	Likert Scale 0-10: Strongly Disagree to

thyroidectomy complications on a real patient in an emergency situation/setting	Strongly Agree. 5=Neutral.
I feel prepared enough to perform this procedure acutely when on-call in an emergency situation	Likert Scale 0-10: Strongly Disagree to Strongly Agree. 5=Neutral.
**STATION 9 (Tonsillectomy)**	
Have you practiced this in a simulated environment before?	Y / N
I am confident to perform a tonsillectomy alone on a real patient in an elective setting	Likert Scale 0-10: Strongly Disagree to Strongly Agree. 5=Neutral.
I am confident to manage a post-tonsillectomy bleed on a real patient in an emergency situation/setting	Likert Scale 0-10: Strongly Disagree to Strongly Agree. 5=Neutral.
I feel prepared enough to perform a tonsillectomy alone electively	Likert Scale 0-10: Strongly Disagree to Strongly Agree. 5=Neutral.
I feel prepared enough to perform an arrest of post-tonsillectomy bleed when on-call in an emergency situation	Likert Scale 0-10: Strongly Disagree to Strongly Agree. 5=Neutral.
**STATION 10 (Front of Neck Access)**	
Have you practiced this in a simulated environment before?	Y / N
I am confident to perform a front of neck access on a real patient in an elective setting	Likert Scale 0-10: Strongly Disagree to Strongly Agree. 5=Neutral.
I am confident to perform a front of neck access on a real patient in an emergency situation/setting	Likert Scale 0-10: Strongly Disagree to Strongly Agree. 5=Neutral.
I feel prepared enough to perform this procedure electively in a clinical setting	Likert Scale 0-10: Strongly Disagree to Strongly Agree. 5=Neutral.
I feel prepared enough to perform this procedure acutely when on-call in an emergency situation	Likert Scale 0-10: Strongly Disagree to Strongly Agree. 5=Neutral.
What do you expect to gain from this bootcamp?	

ENT Southern ST3 Accelerated Learning Course:

POST SESSION-QUESTIONNAIRE

Name for Certificate	
Email Address for Certificate	
Deanery / Hospital	
HST ST3 / Run Through Trainee / Other	
**After attending this bootcamp teaching event, please rate your confidence and preparedness for starting as an ST3 registrar:**	
Do you feel this bootcamp improved your preparedness to undertake your job as an ST3?	
What do you feel you gained from bootcamp to help prepare you for the job or improve your confidence?	
**STATION 1 (Rigid Ventilating Bronchoscopy)**	
I am confident to perform a rigid bronchoscopy on a real patient in an elective setting	Likert Scale 0-10: Strongly Disagree to Strongly Agree. 5=Neutral.
I am confident to perform a rigid bronchoscopy on a real patient in an emergency situation/setting	Likert Scale 0-10: Strongly Disagree to Strongly Agree. 5=Neutral.
I feel prepared enough to perform this procedure electively in a clinical setting	Likert Scale 0-10: Strongly Disagree to Strongly Agree. 5=Neutral.
I feel prepared enough to perform this procedure acutely when on-call in an emergency situation	Likert Scale 0-10: Strongly Disagree to Strongly Agree. 5=Neutral.
How did you find the quality of the teaching at this station?	
How did you find the quality of the simulated environment?	
**STATION 2 (Mastoidectomy)**	
I am confident to perform a cortical mastoidectomy on a real patient in an elective setting	Likert Scale 0-10: Strongly Disagree to Strongly Agree. 5=Neutral.
I am confident to perform a cortical mastoidectomy on a real patient in an emergency situation/setting	Likert Scale 0-10: Strongly Disagree to Strongly Agree. 5=Neutral.
I feel prepared enough to perform this procedure electively in a clinical setting	Likert Scale 0-10: Strongly Disagree to Strongly Agree. 5=Neutral.
I feel prepared enough to perform this procedure acutely when on-call in an	Likert Scale 0-10: Strongly Disagree to Strongly Agree. 5=Neutral.

emergency situation	
How did you find the quality of the teaching at this station?	
How did you find the quality of the simulated environment?	
**STATION 3 (leading a WR)**	
I am confident to lead an inpatient ward round in a hospital setting	Likert Scale 0-10: Strongly Disagree to Strongly Agree. 5=Neutral.
I feel prepared enough to lead ward rounds on inpatients	Likert Scale 0-10: Strongly Disagree to Strongly Agree. 5=Neutral.
Have you been the lead clinician for inpatient ward rounds before?	Y / N
Have you practiced this in a simulated environment before?	Y / N
How did you find the quality of the teaching at this station?	
How did you find the quality of the simulated environment?	
**STATION 4 (NOTSS)**	
How did you find the quality of the teaching at this station?	
How did you find the quality of the simulated environment?	
**STATION 5 (Hololens)**	
I am confident to manage acute ENT conditions in an elective setting	Likert Scale 0-10: Strongly Disagree to Strongly Agree. 5=Neutral.
I feel prepared enough to manage acute ENT conditions electively in a clinical setting	Likert Scale 0-10: Strongly Disagree to Strongly Agree. 5=Neutral.
How did you find the quality of the teaching at this station?	
How did you find the quality of the simulated environment?	
**STATION 6 (SPA ligation)**	
I am confident to perform an SPA ligation on a real patient in an elective setting	Likert Scale 0-10: Strongly Disagree to Strongly Agree. 5=Neutral.
I am confident to perform SPA ligation on a real patient in an emergency situation/setting	Likert Scale 0-10: Strongly Disagree to Strongly Agree. 5=Neutral.
I feel prepared enough to perform this procedure electively in a clinical setting	Likert Scale 0-10: Strongly Disagree to Strongly Agree. 5=Neutral.
I feel prepared enough to perform this procedure acutely when on-call in an emergency situation	Likert Scale 0-10: Strongly Disagree to Strongly Agree. 5=Neutral.
How did you find the quality of the teaching at this station?	
How did you find the quality of the simulated environment?	
How did you find the quality of the teaching at this station?	
How did you find the quality of the simulated	

environment?	
**STATION 7 (Tracheostomy)**	
I am confident to perform a surgical tracheostomy on a real patient in an elective setting	Likert Scale 0-10: Strongly Disagree to Strongly Agree. 5=Neutral.
I am confident to perform a surgical tracheostomy on a real patient in an emergency situation/setting	Likert Scale 0-10: Strongly Disagree to Strongly Agree. 5=Neutral.
I feel prepared enough to perform this procedure electively in a clinical setting	Likert Scale 0-10: Strongly Disagree to Strongly Agree. 5=Neutral.
I feel prepared enough to perform this procedure acutely when on-call in an emergency situation	Likert Scale 0-10: Strongly Disagree to Strongly Agree. 5=Neutral.
How did you find the quality of the teaching at this station?	
How did you find the quality of the simulated environment?	
**STATION 8 (Post Thyroidectomy bleed)**	
I am confident to manage postoperative thyroidectomy complications on a real patient in an emergency situation/setting	Likert Scale 0-10: Strongly Disagree to Strongly Agree. 5=Neutral.
I feel prepared enough to perform this procedure acutely when on-call in an emergency situation	Likert Scale 0-10: Strongly Disagree to Strongly Agree. 5=Neutral.
How did you find the quality of the teaching at this station?	
How did you find the quality of the simulated environment?	
**STATION 9 (Tonsillectomy)**	
I am confident to perform a tonsillectomy alone on a real patient in an elective setting	Likert Scale 0-10: Strongly Disagree to Strongly Agree. 5=Neutral.
I am confident to manage a post-tonsillectomy bleed on a real patient in an emergency situation/setting	Likert Scale 0-10: Strongly Disagree to Strongly Agree. 5=Neutral.
I feel prepared enough to perform a tonsillectomy alone electively	Likert Scale 0-10: Strongly Disagree to Strongly Agree. 5=Neutral.
I feel prepared enough to perform an arrest of post-tonsillectomy bleed when on-call in an emergency situation	Likert Scale 0-10: Strongly Disagree to Strongly Agree. 5=Neutral.
How did you find the quality of the teaching at this station?	
How did you find the quality of the simulated environment?	
**STATION 10 (Front of Neck Access)**	
I am confident to perform a front of neck access on a real patient in an elective setting	Likert Scale 0-10: Strongly Disagree to Strongly Agree. 5=Neutral.
I am confident to perform a front of neck access on a real patient in an emergency situation/setting	Likert Scale 0-10: Strongly Disagree to Strongly Agree. 5=Neutral.

I feel prepared enough to perform this procedure electively in a clinical setting	Likert Scale 0-10: Strongly Disagree to Strongly Agree. 5=Neutral.
I feel prepared enough to perform this procedure acutely when on-call in an emergency situation	Likert Scale 0-10: Strongly Disagree to Strongly Agree. 5=Neutral.
How did you find the quality of the teaching at this station?	
How did you find the quality of the simulated environment?	
**General:**	
What would you have liked if it was done again?	
Any further comments?	
